# Effect of household size on mental problems in children: results from the Norwegian Mother and Child Cohort study

**DOI:** 10.1186/s40359-016-0136-1

**Published:** 2016-06-02

**Authors:** Bjørn Grinde, Kristian Tambs

**Affiliations:** Division of Mental Health, Norwegian Institute of Public Health, Postbox 4404, Nydalen, 0403 Oslo Norway

**Keywords:** Household size, Mental problems, Siblings, Birth order, Evolutionary perspective, Childhood, Social affiliations, MoBa

## Abstract

**Background:**

Most people in industrialized societies grow up in core (parents only) families with few if any siblings. Based on an evolutionary perspective, it may be argued that this environment reflects a mismatch, in that the tribal setting offered a larger number of close affiliates. The present project examined whether this mismatch may have a negative impact on mental health.

**Methods:**

We used data from the Norwegian Mother and Child Cohort Study (MoBa), which includes 114 500 children. The mothers were recruited during pregnancy and followed up with questionnaires as the infants grew older. Correlates between number and type of people living in the household and questions probing mental health were corrected for likely confounders.

**Results:**

The number of household members correlated with scores on good mental health at all ages tested (3, 5 and 8 years). The effects were distinct, highly significant, and present regardless of how mental issues were scored. The outcome could be attributed to having older siblings, rather than adults beyond parents. The more siblings, and the closer in age, the more pronounced was the effect. Living with a single mother did not make any difference compared to two parents. Girls were slightly more responsive to the presence of siblings than boys. Household pets did not have any appreciable impact.

**Conclusion:**

A large household is associated with fewer mental problems in children.

## Background

The high prevalence of anxiety and depression related problems in adolescents and adults suggests that the current environment, or way of life, is not optimal. An evolutionary perspective may help identify possible contributing factors. The concept *Environment of Evolutionary Adaptation* (EEA) has been coined to suggest a type of environment in which we are genetically designed to flourish [1]. While most discrepancies, or mismatches, between the present setting and the EEA are either neutral or beneficiary, some presumably contribute to mental or physical morbidity. These latter may be referred to as *discords* [2, 3]. If we can pinpoint the discords responsible for the high prevalence of mental problems, it may be possible to initiate preventive measures. As the brain is most malleable during the first years of life, it seems reasonably to focus on infancy.

While it is relatively easy to suggest mismatches, it requires dedicated research to identify relevant discords. The point is succinctly exemplified in the case of near-sightedness. The difference in prevalence between people living in cities (up to 80 % in young men) compared to rural areas (typically 1 %) [4] strongly suggests the involvement of discords. The leading candidates, in the form of obvious mismatches, were: one, focusing on a close and fixed distance (as in reading); and two, not having a natural diurnal cycle of light (the light being on at night). However, recent research suggests that the main discord is the lack of time infants spend outdoor, as the eyes require a certain amount of strong (sun)light in order to develop correctly [5].

Mental problems are considerably more difficult to deal with than near-sightedness, consequently it is particularly important to find relevant discords. If the environment can be adapted accordingly, it may reduce the future toll of mental agony.

As in the case of near-sightedness, there is a range of candidate discords, particularly in connection with anxiety [6]. Data from the Norwegian Mother and Child Cohort Study (MoBa) offer an opportunity to investigate some factors [7].

The MoBa questionnaires were not designed with an evolutionary perspective in mind, and are therefore not ideal for the present purpose. Moreover, the Norwegian population is relatively homogenous as to key child rearing practices, and thus not suitable for the evaluation of all potential discords. We consequently focused on one factor: the number of people present in the household. There is appreciable variation in the MoBa cohort as to household size. Information regarding age-classified members is gathered during pregnancy, implying that the siblings recorded are older than the index child. In a typical (Stone Age) tribal setting, there would be a larger number of close affiliates compared to typical homes in industrialized societies. The affiliates would presumably offer a sense of safety as well as a social context, input that theoretically could reduce the activation of fear and low mood modules of the brain. Less activation would mean less “exercise” of these functions, and thus less strengthening of the underlying neural circuits. In other words, a perceived lack of company by supportive people could be theorized to increase the risk of both anxiety and depression related problems.

A strong social network is well known to contribute to well-being, mental health and longevity in adults [8–10], including young adults [11]. For infants, the family constitutes the main social context. The question is whether a similar effect can be seen in infants, and if so, which relatives or others contribute in this direction.

There are some previous reports investigating the relationship between mental problems and the size of family in which infants grow up, primarily looking at effects later in life. In a deprived setting, the correlation may actually be positive; i.e., a large number of siblings have a negative impact. For example, a study of poor, rural communities in Mexico found that family size predicts anxiety in adolescents [12]; and a related study of urban slum-children in India found a correlation between family size and psychiatric disorders [13]. However, these results may reflect the stress and problems related to raising many children with limited resources.

Data from Western, affluent settings are more conflicting. A UK based study found a univariate association between family size and increased risk of childhood psychiatric disorder, but the association disappeared when correcting for confounders [14]. In this study, siblings were recorded as either “2 or fewer”, or “3 or more”. Socieconomic factors appeared more important than the number of siblings. Another UK study suggests that having one or two siblings may be protective, while larger families may cause an increased chance of mental problems in the elder siblings [15]. Data from China indicate reduced depression in adolescents who did not have any siblings [16], but the one child family policy in this country may contribute to the result.

In these studies, the outcomes are mental problems of sufficient magnitude to warrant a diagnosis at later stages in life. A report from Australia was based on a design more similar to the one used in the present study [17]. They used questionnaires filled in by the mothers, and found that small family size predicts internalizing behaviour in infants. The present study was also based on mothers’ reports on their infants while they were still young. The questionnaires used in both studies indicate internalising (anxiety, depression) and externalising (aggression, opposition, defiance) behaviour. A high score on these instruments predicts mental health issues later in life [18, 19].

The present study included a number of variables regarding the type of household the infant was born into. Thus, the dataset allowed for the adjustment for key confounders such as maternal age, maternal and paternal educational level, family income, maternal and paternal period of leave from work after birth, maternal breastfeeding status when child is 18 months, and the presence of pets. We aimed to examine the possible effects of: 1) family size in general; and 2) type of members (one or two parents, grandparents, other adults, siblings). The outcome variables were: 1) temperament; 2), behaviour problems; and 3) symptoms of anxiety and depression. The scores were obtained for the age period 3-8 years. As the information was registered for new-born infants, the dataset only include information as to older siblings. The data was not suitable for the question of whether younger siblings would give a similar effect.

## Methods

### Norwegian Mother and Child Cohort Study

The Norwegian Mother and Child Cohort Study (MoBa) (http://www.fhi.no/morogbarn) is a prospective, population-based cohort study initiated by the Norwegian Institute of Public Health [7, 20]. Participants (95 200 mothers and 114 500 children) were recruited from throughout Norway from 1999 to 2008 and are almost exclusively ethnic Norwegians. Participants did not receive financial compensation, yet 40.6 % of those approached were enrolled. A written informed consent was obtained from participants, as well as a licence from the Norwegian Data Inspectorate. There are follow-ups with new questionnaires at regular intervals. Using data from the Medical Birth Registry of Norway, it has been indicated that although prevalence estimates of exposures and outcomes in the MoBa study may be biased owing to selection, estimates of exposure-outcome associations are less likely to be affected, and therefore do not constitute a serious validity problem in terms of representativeness [7].

The present study is based on version 8 (released February 2014) of the quality-assured data files. The study has been approved by the Regional Committee for Medical Research Ethics. Data were collected from Questionnaire 1 (gestational week 17), Questionnaire 4 (6 months after birth), Questionnaire 5 (18 months after birth), Questionnaire 6 (3 years after birth), Questionnaire 7 (5 years after birth), and Questionnaire 8 (8 years after birth). The dependent variables were based on responses from mothers of respectively 51 569 children (3 years), 28 627 children (5 years), and 17 594 children (8 years). The reduction in numbers, compared to the initial recruitment, is due to the combination of: 1) A general tendency to drop out as the child ages; 2) lack of response to key questions; 3) the questionnaire was only sent to a subset of parents (5 years); and 4) the participants were recruited over a 10 year period, and the part of the sample recruited most recently had not completed the last questionnaire (8 years).

The present study focused on household size as the main exposure variable. Questionnaire 1, which was submitted during pregnancy, asks about the number of persons sharing the household. There were also more detailed questions about types of relatives in the household. These data were used to probe for correlates to questions relating to mental issues in the children as they grew older.

### Measures

#### Independent variables

*Household size* and *type* were reported in Questionnaire 1 (during pregnancy). The mothers responded to items such as: “How many people, including you, live in your home?”, and “With whom do you live?”. Response categories were “Spouse/partner”, “Parents”, “Parents-in-law”, “Children”, “No one”, and “Other, describe”. Number and approximate age of children (presumed to be siblings) present (not including the study child) was reported as: Number of people between: “12–18 years”, “6–11 years”, and “under 6 years”, respectively. The numbers of siblings were coded 1, 2, or >2.

#### Covariates

Some variables were included because they might confound the relationship between household size and children’s mental health: *Maternal and paternal period of leave from work after child’s birth*, was reported by the mother for herself and for the child’s father when the child was 18 months. Maternal leave was coded: no leave = 0, <2 months = 1, 2–6 months = 2, 6–9 months = 3, 9–12 months = 4, 12–18 months = 5, >18 months = 6. Paternal leave was coded: no leave = 0, <1 month = 1, 1–2 months = 2, 2–3 months = 3, 3–6 months = 4, and >6 months = 5. Duration of *breastfeeding* was reported when the child was 18 months. A summative index was generated and scored 0–5 based on whether the mother reported breastfeeding at: 6–8 months = 1, 9–11 months = 2, 12–14 months = 3, 15–18 months = 4. The last category also included those who reported to be breastfeeding at least once a week at 18 months. The presence of *Animals* (pets) in the family, reported when the child was 6 months, was coded as a dichotomous variable. The data were adjusted for a number of additional covariates: *Maternal age* was categorized into five year intervals from <20 years to ≥35 years. *Maternal and paternal income* (reported in Questionnaire 1) were summed and used as a categorical variable with seven response categories from no income to > NOK 500 000. *Maternal and paternal education* were reported in the same questionnaire as one of six categories ranging from: “9 year elementary school” to “at least 4 years at university”. *The child’s sex* was also used as a covariate.

#### Dependent variables

The outcome variables were generated based on symptoms at age 3, 5 and 8 years as reported by the mother. The items at age 3 and 5 were picked from various symptom lists in the questionnaires, based on what the authors judged as good face validity for the present purpose.. The data were factor analysed using an oblique rotation. In the 3 year questionnaire, we used all the 26 available items from the *Child Behavior Checklist* (CBCL) [21]. Four factors were generated: *Emotional regulation*, *Anxiety*, *Eating/somatic*, and *Hyperactivity/concentration*. Items and the factor loadings are shown in Table 1.

**Table 1:** Factor loadings for the items included (bold) in the outcome measures at age 3 years

	Emotional regulation	Anxiety	Eating/somatic	Hyperactivity/concentration
Demands must be met immediately	**.70**	.27	.13	.18
Defiant	**.65**	.15	.13	.10
Gets in many fights	**.62**	.08	.10	.23
Can’t stand waiting. wants everything now	**.62**	.25	.10	.36
Gets into everything	**.52**	.13	.09	.26
Hits others	**.49**	.02	.08	.21
Resists going to bed at night	**.42**	.13	.36	-.08
Punishment doesn’t change his/her behaviour	**.42**	.13	.23	.39
Sudden changes in moods and feelings	**.41**	.25	.25	.26
Too fearful or anxious	.10	**.64**	.28	.09
Afraid to try new things	-.03	**.60**	.14	.08
Clings to adults or are too dependent	.30	**.59**	.21	.13
Disturbed by any change in routine	.27	**.55**	.15	.16
Gets too upset when separated from parents	.16	**.54**	.19	.03
Doesn’t eat well	.17	.13	**.75**	.13
Doesn’t seem to be happy eating food (exc. sweets)	.13	.11	**.71**	.23
Stomach aches or cramps (without medical cause)	.14	.20	**.43**	.06
Doesn’t want to sleep alone	.31	.23	**.37**	-.18
Constipated, doesn’t move bowels	.02	.19	**.36**	-.01
Vomiting, throwing up (without medical cause)	.01	.14	**.28**	.13
Can’t concentrate, can’t pay attention for long	.25	.16	.11	**.72**
Can’t sit still, restless or overactive	.34	.09	.12	**.67**
Quickly shifts from one activity to another	.39	.12	.17	**.53**
Poorly coordinated or clumsy	.00	.33	.13	**.35**
Doesn’t seem to feel guilty after misbehaving	.14	.09	.11	**.33**
Eats and drinks things that are not food (not sweets)	.16	.10	.16	**.31**

Note: Loadings are from the structure matrix, oblique rotation. Intercorrelations between the factor scores range from to

Outcome variables at 5 years of age were based on nine selected items from the CBCL, five from the *Emotionality, Activity and Shyness Temperament Questionnaire* (EAS) [22], and two items made for the MoBa study. Data from these 16 items were factor analysed with an oblique rotation. A two factor solution was chosen. The first factor was referred to as *Anxiety*, the other *Difficult temperament*. Items and factor loadings are shown in Table 2.

**Table 2:** Factor loadings for the items included (bold) in the outcome measures at age 5 years

	Anxiety	Difficult temperament
EAS: Your child takes a long time to warm up to strangers	**-.69**	.05
EAS: Your child is very friendly with strangers	**.59**	.04
CBCL: Too fearful or anxious	**.57**	-.35
CBCL: Afraid to try new things	**.55**	-.21
Avoids to talk to others than family members	**.54**	-.09
CBCL: Gets too upset when separated from parents	**.52**	-.24
CBCL: Nervous, high strung and tense	**.51**	-.41
CBCL: Clings to adults or too dependent	**.51**	-.38
CBCL: Fears certain animals, situations or places	**.44**	-.18
CBCL: Disturbed by any change in routine	**.43**	-.39
Had following problems: Emotional difficulties (sad and worried)	**.32**	-.27
EAS: Your child gets upset or sad easily	-.20	**.82**
EAS: Your child cries easily	-.24	**.74**
EAS: Your child reacts intensely when upset	-.13	**.68**
CBCL: Cries a lot.	.26	**-.67**
CBCL: Unhappy, sad or depressed	.30	**-.39**

Note: Loadings are from the structure matrix, oblique rotation. The correlation between the factor scores is 0.32

The 8 year questionnaire included short-forms of two instruments: The *Screen for Child Anxiety Related Emotional Disorders* (SCARED) [23] is a multidimensional instrument generated to measure Diagnostic and Statistical Manual of Mental Disorders (DSM)-defined anxiety symptom in children. The present *Anxiety* score was based on a five item version [24]. The response categories were “Not true”, “Sometimes true” and “True”. Our *Depression* score was based on the 13 item version of the DSM-adapted *Short Mood and Feelings Questionnaire* (SMFQ) [25]. The response categories were the same as in SCARED. The list of items is shown in Table 3.

**Table 3:** The 13 item version of the Short Mood and Feeling Questionnaire

1. Felt miserable or unhappy
2. Felt so tired that s/he just sat around and did nothing
3. Was very restless
4. Didn’t enjoy anything at all
5. Felt s/he was no good anymore
6. Cried a lot
7. Hated him/herself
8. Thought s/he could never be as good as other kids
9. Felt lonely
10. Thought nobody really loved him/her
11. Felt s/he was a bad person
12. Felt s/he did everything wrong
13. Found it hard to think/concentrate

In addition to the mental health outcomes, we used a number of questions on somatic outcomes for supplementary analyses. The purpose was to test for possible confounding effects of maternal temperament. A worried, or particularly attentive, mother might report more symptoms of both mental and somatic nature (see the description of statistical analyses). An index of somatic health problems was compiled based on the 3 and 5 year Questionnaires. A score of 1 was given for long-term issues during either the first 18 months or 18–36 months. The list included: impaired hearing, impaired vision, delayed motor development, joint problems, gained too little weight, gained too much weight, asthma, allergy affecting eyes or nose, eczema, food allergy/intolerance, gastrointestinal problems, late or abnormal speech development, and other long-term illness or health problems. Another index was based on short-term illness reported at age 3, including: ear infection, bronchitis, gastric flu/diarrhoea, injury or accident. A third index included health problems reported when the child was 5: asthma, pollen allergy/hay fever, obstruction/wheezing in chest, impaired hearing, delayed motor development or clumsy, delayed or deviant language development, impaired vision, or other health problem. The three indices were analysed separately and summed to one general index. Although several factors could conceivable contribute to correlations between somatic and mental problems, the comparison should help clarify the issue of reporting bias.

All the dependent variables were transformed to z-scores in order to obtain estimates with easily interpretable effect sizes.

### Missing values

The *outcome variables* on children’s mental health were imputed with the Statistical Package for the Social Sciences (SPSS) EM imputation procedure, where correlated valid data are used to predict values replacing missing values. Each of the sets of items was imputed separately in cases where at least half the items had valid data. Data sets with more than 50 % missing data were discarded. The variables relating to household size and type, animals in the family, as well as somatic health were based on checking or not checking a number of categories. As there were no contra-categories (no place to check for “no”), there were no missing data for these variables. Maternal and paternal period of leave from work after child’s birth were entered in the analyses as categorical variables, and missing data were recoded to separate categories. Missing breastfeeding data were recoded to the lowest category. Results from cross tabulations of the highly correlated variables *maternal and paternal educational level*, suggested that missing data should be categorized together with the lowest category. Based on similar reasoning, missing data on *family income* were recoded to the second lowest income group.

### Statistical analyses

We estimated the association between the principal predictor and the outcome variables using a variance analysis procedure, *SPSS Generalized Linear Models*. In our first set of analyses, household size was specified as a factor together with categorical maternal age, categorical maternal breastfeeding duration, and pets in the family. Maternal and paternal duration of leave after birth, maternal and paternal educational levels, and family income were entered as linear covariates. Each of the eight outcome variables (four at age 3, two at age 5 and two at age 8) were consecutively used as dependent variable. In a second series of analyses, household size was replaced with variables specifying numbers of various types of relatives in the family: 1) Spouse of mother (usually the child’s father), 2) parents of mother, 3) parents of spouse (parents in law), 4) children (siblings of the index child), and 5) others. Only the outcome variables with the strongest association with household size in the first set of analyses were included in the second set. In a third set of analyses possible age specific effects of siblings in the household were examined, entering three separate variables for number (0, 1, or 2+) of older siblings, aged 0–5 years, 6–11 years, and 12–18 years at pregnancy, respectively. There were significant interaction effects between household size and sex of the child, and as a final step the analyses were conducted stratified by sex.

The outcome variables were reported by the mothers, and will to some extent be affected by individual judgement. Among other factors, maternal concern and worriedness for her child might affect the outcome scores. Worried mothers would presumably be more likely to judge signs in their children as negative symptoms. If maternal concern about her child varies systematically with the number of children, for instance if earlier experience with being a mother makes her safer and more relaxed, the mothers’ concern might confound a possible association between birth order and mental health. To the extent that this was the case, one would expect the concern to generalize to somatic health problems. That is, inexperienced mothers would also tend to judge their children’s somatic health as worryingly. To examine such a possible confounding, three general indices of “maternally perceived somatic health problems in the child” were generated. The items included were selected based on whether the responses were likely to depend on personal judgement, and thus be affected by maternal concern. The indices were also relevant as a gauge for other factors that may vary systematically with parity. Thus, the analyses were repeated with the somatic indices as outcome in order to indicate the extent to which such a bias may have affected our results on mental health.

## Results

Factor loadings for the various outcome variables at 3 and 5 years are shown in Tables 1 and 2. By including only the items which had their strongest loading on a specific factor, we (conservatively) estimated the alpha reliability for that factor. For instance, the estimate for Emotional regulation at age 3 was based on data from the upper nine items in Table 1. We obtained the following alpha reliabilities for the measures at age 3: 0.73 (Emotional regulation), 0.55 (Anxiety), 0.46 (Eating/somatic), and 0.54 (Hyperactivity/concentration). The values for 5 years were: 0.72 (Anxiety) and 0.71 (Difficult temperament). Values for the instruments used at 8 years were 0.45 (Anxiety) and 0.89 (Depression).

As detailed in the Methods section, the study examined correlates between household composition and symptoms of poor mental health in children. The results presented have been adjusted for the following factors considered to be potential confounders: maternal age, maternal and paternal educational level, family income, maternal and paternal period of leave from work after birth, maternal breastfeeding status when child is 18 months, and animals living with the family. Some of the adjustments did lower the estimated effect sizes, but they did not drastically affect the significance of the results.

In Table 4, the exposure is categorized as to the total number of people present in the household. The value “1” implies that the mother is single, while “2” usually means a couple without any previous children. The latter score was used as a reference. Higher numbers reflect a combination of older siblings and adult relatives. The presence of more than parents had a protective effect on the child (a negative score implies a reduced tendency to have mental problems) regardless of the type of outcome. The results are shown as fractions of standard deviations of the outcome variables compared to the reference group (two persons). The association between household size and child mental health was highly significant, *p* < 10^−9^ for all outcomes. There was a distinct tendency for larger households to yield more pronounced results, the effect size reaching -0.39 of for household sizes of 6 or more. The outcome included both typical internalizing problems (anxiety and depression) and problems related to externalizing behaviour (hyperactivity, difficult temperament). Being a single mother did not significantly affect the child’s mental health.

**Table 4:** Adjusted mean scores (M) with confidence intervals (CI) of mental health related problems by household size

Household size	N^a^	3 years				5 years		8 years	
		Emotional regulation	Anxiety	Eating/somatic	Hyperactivity/concentration	Anxiety	Difficult temperament	Anxiety	Depression
		M (95 % CI)	M (95 % CI)	M (95 % CI)	M (95 % CI)	M (95 % CI)	M (95 % CI)	M (95 % CI)	M (95 % CI)
1	1 598	.00 (-.05, .05)	-.04 (-.10, .01)	.04 (-.02, .09)	-.01 (-.06, .04)	-.03 (-.10, .05)	.05 (-.03, .12)	.01 (-.09, .10)	.11 (-.01, .21)
2	23 535	-	-	-	-	-	-	-	-
3	17 041	.02 (.00, .04)	-.24 (-.26,-.22)	-.23 (-.25,-.21)	-.08 (-.10,-.06)	-.15 (-.17,-.12)	-.13 (-.16,-.10)	-.10 (-.14,-.07)	-.08 (-.11,-.04)
4	7388	-.07 (-.10,-.04)	-.29 (-.31,-.26)	-.25 (-.28,-.22)	-.24 (-.27,-.21)	-.22 (-.25,-.18)	-.31 (-.35,-.28)	-.16 (-.20,-.11)	-.14 (-.18,-.10)
5	1 528	-.15 (-.20,-.10)	-.30 (-.35,-.25)	-.28 (-.33,-.23)	-.28 (-.33,-.23)	-.16 (-.24,-.09)	-.29 (-.36,-.23)	-.20 (-.28,-.11)	-.12 (-.20,-.04)
6+	479	-.09 (-.19,-.00)	-.33 (-.42,-.24)	-.23 (-.32,-.14)	-.25 (-.33,-.16)	-.27 (-.38,-.16)	-.39 (-.50,-.28)	-.24 (-.39,-.08)	-.10 (-.26, .07)

^a^Numbers of participants is for 3 years of age. Approximate numbers are 56 % of the listed figures for 5 years, and 34 % for 8 years

The outcome scores are z-scaled (SD = 1) with parents only (size = 2) as reference. The results are adjusted for maternal age, maternal and paternal educational level, family income, maternal and paternal period of leave from work after child’s birth, maternal breastfeeding status when child is 18 months, and animals in the family. *p* < 10^−9^ (overall test of mean differences between categories) for the effect of household size (3 and more) on all outcome variables

Further analyses were performed in order to elucidate the nature of the observed effect, focusing on the outcome variables showing the strongest effect in Table 4.

The associations between mental health and types of relatives present in the household were examined. Table 5 shows specific effects of the presence of various types of relatives, each included as separate predictors in the multivariate analysis. The results demonstrate that the effect of family size was primarily driven by the presence of siblings. There were no indications that the presence of additional adults improved child behaviour, except for a just-significant protective effect of spouse (usually father of the child) on difficult temperament. The only other significant effect was an increased tendency of difficult temperament in five year old children in the presence of “others” (not children, parents or grandparents).

**Table 5:** Adjusted mean scores (M) with confidence intervals (CI) of mental health related problems by types of relatives in the household

Types of relatives in the household	N^a^ Tot = 51 569	3 years		5 years		8 years
		Anxiety	Hyperactivity/concentration	Anxiety	Difficult temperament	Anxiety
		M (95 % CI)	M (95 % CI)	M (95 % CI)	M (95 % CI)	M (95 % CI)
Spouse of mother	49 859	-.02 (-.11, .05)	-.01 (-.08, .06)	-.06 (-.13, .02)	**-.11 (-.21,-.02)**	.01 (-.07, .10)
Parent(s) of mother	504	.04 (-.06, .14)	.00 (-.10, .09)	.01 (-.14, .16)	.00 (-.14, .15)	-.08 (-.27, .11)
Parent(s) in law	223	.13 (-.03, .28)	-.03 (-.16, .10)	.02 (-.18, .23)	.01 (-.18, .20)	-.01 (-.21, .18)
Children^b^	21 944	**-.23 (-.25,-.21)**	**-.11 (-.13,-.09)**	**-.14 (-.16,-.11)**	**-.15 (-.18,-.13)**	**-.09 (-.12,-.06)**
Others	1 087	.03 (-.03, .09)	-.02 (-.08, .04)	-.04 (-.12, .04)	**.10 (.01, .18)**	.06 (-.05, .18)

^a^Total number of participants is for 3 years of age. The corresponding sample sizes are 28 627 for 5 years and 17 594 for 8 years

^b^Usually siblings of the child participating in the study

The outcome scores are z-scaled (SD = 1). Each row represents separate dichotomous variables, a subject may have checked for none, some, or all. The effects of each of the variables in the Table were adjusted for each other, as well as for maternal age, maternal and paternal educational level, family income, maternal and paternal period of leave from work after birth, maternal breastfeeding status when child is 18 months, and animals in the family. Significant effects (*p* < 0.05) in bold

The above results prompted the investigation of whether the age of siblings mattered. It should be noted that the questionnaires were filled in prior to the birth of the child being examined, thus the actual age of siblings would be higher during the period of exposure. Moreover, some of the children would eventually obtain younger siblings, of which there is no available information. As shown in Table 6, the results were consistent with the finding that the more siblings the better; but the best scores were obtained with siblings not too different in age. Again the effect was observed regardless of the way mental health was evaluated.

**Table 6:** Adjusted mean scores (M) with confidence intervals (CI) of mental health related problems by age category of siblings

Age/number of older siblings		N^a^ Tot = 51 569	3 years		5 years		8 years
			Anxiety	Hyperactivity/concentration	Anxiety	Difficult temperament	Anxiety
			M (95 % CI)	M (95 % CI)	M (95 % CI)	M (95 % CI)	M (95 % CI)
<6 years	0	28 789	-	-	-		
	1	19 239	**-.25 (-.26,-.23)**	**-.08 (-.10,-.07)**	**-.16 (-.18,-.13)**	**-.15 (-.18,-.13)**	**-.10 (-.14,-.07)**
	2+	3 541	**-.39 (-.42,-.36)**	**-.27 (-.30,-.24)**	**-.27 (-.31,-.22)**	**-.34 (-.39,-.30)**	**-.19 (-.24,-.14)**
6–11 year	0	44 317	-	-	-	-	-
	1	5 473	**-.06 (-.09,-.03)**	**-.15 (-.18,-.12)**	**-.05 (-.09,-.01)**	**-.19 (-.22,-.15)**	**-.14 (-.19,-.10)**
	2+	1 779	**-.18 (-.23,-.14)**	**-.24 (-.29,-.19)**	**-.13 (-.20,-.07)**	**-.33 (-.39,-.27)**	**-.14 (-.21,-.06)**
12–18 years	0	49 273	-	-	-	-	-
	1	1 777	-.02 (-.07, .03)	-.04 (-.09, .01)	-.04 (-.11, .03)	-.03 (-.10, .04)	-.02 (-.11, .07)
	2+	519	-.08 (-.16, .00**)**	-.08 (-.17, .01)	-.06 (-.17, .05)	**-.11 (-.22,-.01)**	.07 (-.08, .23)

^a^Total number of participants is for 3 years of age. Overall sample sizes are 28 627 for 5 years and 17 594 for 8 years

The outcome scores are z-scaled (SD = 1) with no siblings of the indicated ages as reference. The results are adjusted for maternal and paternal educational level, family income, maternal and paternal period of leave from work after birth, maternal breastfeeding status when child is 18 months, and animals in the family. Significant effects (*p* < 0.05) in bold

Another question was whether the child’s sex made a difference. We tested “sex x household size” interaction effects by adding interaction terms to the initial analyses (the results from which were shown in Table 4). The interaction effect reached significance (*p* < .01) for four outcome variables. New analyses of these outcomes were stratified by sex, as displayed in Table 7. The results show somewhat stronger effects of household size for girls than for boys.

**Table 7:** Adjusted mean scores (M) with confidence intervals (CI) of mental health related problems by household size, stratified by sex

Household size	3 years				5 years		8 years	
	Eating/somatic		Hyperactivity/concentration		Difficult temperament		Anxiety	
	Boys	Girls	Boys	Girls	Boys	Girls	Boys	Girls
1	.02 (-.06, .10)	.04 (-.04, .12)	.01 (-.06,. 09)	-.02 (-.10, .05)	.09 (-.14, .20)	.01 (-.10, .12)	.06 (-.07, .20)	-.05 (-.17, .07)
2	-	-	-	-	-	-	-	-
3	-.23 (-.26,-.20)	-.23 (-.26,-.30)	-.08 (-.11,-.06)	-.10 (-.12,-.07)	-.12 (-.15,-.08)	-.14 (-.18,-.11)	-.14 (-.19,-.09)	-.06 (-.11,-.01)
4	-.24 (-.28,-.20)	-.26 (-.30,-.22)	-.24 (-.28,-.20)	-.25 (-.28,-.21)	-.28 (-.33,-.24)	-.33 (-.38,-.28)	-.16 (-.22,-.10)	-.15 (-.21,-.09)
5	-.26 (-.33,-.19)	-.31 (-.38,-.23)	-.28 (-.35,-.21)	-.30 (-.37,-.23)	-.22 (-.31,-.12)	-.38 (-.48,-.28)	-.18 (-.29,-.07)	-.21 (-.33,-.08)
6+	-.25 (-.37,-.13)	-.23 (-.36,-.10)	-.22 (-.34,-.09)	-.33 (-.45,-.21)	-.38 (-.52,-.24)	-.41 (-.58,-.25)	-.15 (-.36,-.07)	-.31 (-.53,-.10)

The outcome scores are z-scaled (SD = 1) with parents only (size = 2) as reference. The results are adjusted for maternal age, maternal and paternal educational level, family income, maternal and paternal period of leave from work after birth, maternal breastfeeding status when child is 18 months, and animals in the family. Results are shown for the outcome variables for which a significant (*p* < .01) overall “sex X household size” interaction effect could be demonstrated; that is, comparing household size = 2 with larger households. *p* < 10^−5^ (overall test of mean differences between categories) for the effect of household size (3 and more) on all outcome variables in both genders

There was no consistent effect of breastfeeding across the various outcomes. A significant positive association for one outcome variable is consistent with a selection effect, where children with emotional difficulties tend to be weaned later than emotionally stable children. There was a consistent but weak trend of protective effect of long maternal leave after birth, reaching significance in three of the outcome variables. There was no consistent effect of paternal leave. There were significant effects of the presence of animals, but pointing in both directions, and with trivial effect sizes.

An analysis using reported somatic problems as outcome found no appreciable effects of having older siblings (Table 8). Out of 24 estimates, five reached significance, but only at *p* < .05. Four of these were positive, suggesting a slight *negative* effect on health. This is in the opposite direction of what was expected based on the hypothesis of a negative relationship between maternal concern and number of earlier born children.

**Table 8:** Adjusted mean scores (M) with confidence intervals (CI) of somatic health problems by age category of siblings

Age/number of older siblings		N^a^ Tot = 51 947	3 years		5 years	3 + 5 years
			Short-term somatic problems	Long-term somatic problems	Somatic problems	Somatic problems
			M (95 % CI)	M (95 % CI)	M (95 % CI)	M (95 % CI)
<6 years	0	28 994	-	-	-	
	1	19 384	**.03 (.01, .05)**	**.07 (.05, .09)**	**.04 (.01, .07)**	**.05 (.03, .08**)
	2+	3 569	.01 (-.01, .05)	-.02 (-.05, .02)	-.02 (-.07, .03)	-.01 (-.06, .04)
6–11 year	0	44 625	-	-	-	-
	1	5 520	.02 (-.01, .05)	.00 (-.03, .03)	.00 (-.04, .04)	.01 (-.03, .06)
	2+	1 802	-.01 (-.05, .04)	**-.06 (-.11,-.01)**	-.05 (-.11, .01)	-.05 (-.12, .02)
12–18 years	0	49 622	-	-	-	-
	1	1 798	.01 (-.04, .06)	.01 (-.05, .05)	-.02 (-.09, .04)	-.01 (-.09, .06)
	2+	527	-.08 (-.16, .01)	.07 (-.03, .17)	.06 (-.06, .18)	.08 (-.07, .23)

^a^Numbers in the full sample of participants at 3 years of age. Overall sample sizes are 28 768 for 5 years and 24 982 for 3 + 5 years

The outcome scores are z-scaled (SD = 1) with no siblings of the indicated ages as reference. The results are adjusted for maternal and paternal educational level, family income, maternal and paternal period of leave from work after birth, maternal breastfeeding status when child is 18 months, and animals in the family. Significant effects (*p* < 0.05) in bold

## Discussion

The purpose of the present study was to identify possible causes of mental problems. The choice of parameters to be examined was based on an evolutionary perspective of the human brain. The strategy implies looking for *mismatches*, in the form of differences between present way of life and the presumed way humans are “genetically designed” to live. Some of the mismatches, referred to as *discords*, may help explain the prevalence of mental problems [2, 3].

It is likely that the Stone Age tribes had more close affiliates for the child to interact with on a continuous basis, compared to what is typically the case in industrialized societies. Although kindergartens offer company, this is only for a limited period of the day, and the kids are not expected to be as closely knit as those brought up in the same family or tribe. As pointed out elsewhere [26], caretaking of infants by siblings (or additional adults) is typical for tribal people. According to the author, the point is reflected in improved life prospective for infants with older siblings. The question is whether this mismatch also qualifies as a discord; that is, does it affect the mental health of children (and thus potentially adults) in industrialized societies?

The present results suggest so. Having older siblings correlated with improved scores on mental outcome for all age groups probed (3, 5 and 8 years), regardless of how the outcome was measured (Tables 4). It is important to emphasise that the figures presented were corrected for obvious confounders such as socioeconomic status, education, and age of mothers. Although the results were highly significant (*p* < 10^−9^), it should be pointed out that the effect only explains a small part of the variation. Perhaps somewhat surprisingly, having a single mother did not appear to be a disadvantage compared to having two parents without older siblings. It should be noted that single mothers in Norway have better conditions than those in many other countries, in terms of governmental support and lack of social stigma.

The observed symptoms are known to predict poor adult mental health [18, 19], but the present results do not tell whether the reported effect will persist. The MoBa project continues, so the answer to that question will hopefully be available in the future.

One possible explanation for the effect is based on how the human brain is designed to be moulded by the environment – particularly in infants. Functions that are frequently activated tend to “expand” and become stronger. Thus, if fear or low mood is often activated during infancy, the results may be excessive activity of these functions later in life, which in the present vocabulary corresponds to problems related to respectively anxiety and depression. As reasoned elsewhere [3], conditions that cause fear in infants include less proximity of care persons and other close affiliates. Older siblings would be expected to supplement parents in terms of offering the child an environment that induces the feeling of safety and companionship. The data were not informative as to whether younger siblings would offer a similar protective effect, although it seems fair to hypothesis that any siblings may do. However, older siblings may be more valuable than having younger siblings, as the latter would presumably add less to the perceived safety. In the EEA, children would presumably grow up with not only siblings, but agemates from other families as well. The total number of children in a group was presumably was most likely considerably larger than what is found in the average family of today.

Parental investment theory offers another interesting angle on the results. The theory suggests a possible conflict between parents and offspring, in that while the parents’ genes are best served by a large number of progeny, the genes of the individual infant are best served by few siblings – in that the latter implies more parental attention and resources [27]. Whether there is a conflict depends, however, on the circumstances [28]. The theory predicts more sibling conflict in families with many children, which is indicated by a report finding that the amount of sibling aggression correlates with family size [29]. However, as to mental health, rivalry could either promote internalizing and externalising behaviour, or build resilience. Moreover, the aggression would typically be sporadic and relatively benign; thus in sum, the effect of interacting with siblings might be positive despite of occasional quarrels. That is, as long as there are ample resources to care for all the children, which is the case in affluent societies such as Norway. The present results support the above contention.

The results could also be described as a correlate between birth order and internalizing/externalizing behaviour. There are several previous reports on birth order effects, but not much in terms of effect on mental health. The more solid observations imply a modest effect on intelligence in that first born Norwegian [30] and Swedish [31] men obtain higher scores. This effect may relate to the older child taking responsibility for younger siblings, and consequently becomes more ambitious or conscientious. The observation does not conflict with the present findings.

Theoretically, one might expect that the presence of adults would be as important as the presence of siblings. According to the data (Table 5), they are not. Additional adults are relatively rare in Norwegian households. Their presence may correlate with family problems that we could not adjust for, and they may be less present in the child’s immediate surroundings compared to older siblings. Moreover, it was interesting to note that the main effect was observed with siblings only slightly older than the child being investigated (Table 6), suggesting that the optimal situation is to have play mates. From an evolutionary viewpoint, it should be mentioned that long term cooperation relies primarily on age mates, thus social affiliations should be tuned toward those of roughly the same age.

Animals may substitute for humans by being companions. Previous reports suggest they may have a positive effect on mental health [32, 33]. Questionnaires 4 and 5 asked the mother whether there were pets in the household as a dichotomous variable. In the present data, we found no appreciable effects of pets (data not shown).

## Limitations

We have interpreted the present results within the theoretical framework of evolutionary adaptation; that is, having few close affiliates is a discord in the sense that it contributes to mental problems by triggering brain functions related to anxiety, loneliness, or lack of social comfort. We cannot, however, exclude alternative explanations. For example, parents who decide to have more than one or two children may be fonder of children and thus offer better child care; or those whose first infant(s) was emotionally stable are more likely to opt for additional offspring. It is also conceivable that mothers (and fathers) of small families have a higher tendency toward anxiety or related mental issues, and pass these traits on either by genetic inheritance or by their way of handling infants. Furthermore, it is unclear whether the effect requires the presence of older siblings, as opposed to infants younger than the index child.

The validity of our outcome measures may also be questioned. Some of the internal consistency reliability estimates are low, but that may be because short version instruments like SCARED, give the best validity and measurement prediction if they sample different types of symptoms, all being criteria of a group of disorders. SCARED was initially generated as a multidimensional anxiety measure, in such a case internal consistency coefficients underestimate the reliability. To the extent that some of the low alpha values really reflect measurement error, this has attenuated the effect estimates of the presence of siblings, meaning that the real effects are even stronger than shown by our results. Also the validity of some of our measures is undocumented and rests on the actual content of the single items (“face validity”). The results are similar for all eight outcomes, however, and the risk that all of them are very poor measures of mental health is minimal.

A major limitation is that all the outcome measures depend on maternal judgement of the child behaviour. Beyond causing imperfect validity and reliability, which is already addressed, we were afraid maternal report would systematically bias the result because the mothers might tend to judge their first child different from later ones. However, there was no appreciable bias as to the mothers’ judgement of somatic health (Table 8).

Although the above caveats are relevant, they seem unlikely to alone explain the observed effects.

## Conclusions

According to the present study, living in a family with older siblings – who offer an opportunity for play, comfort and security – protects against developing internalizing and externalizing behavioural problems. The effect is distinct and highly significant. In a world suffering from overpopulation, it is not obvious how the observation should be incorporated in governmental advice.

## Abbreviations

CBCL, Child Behavior Checklist; DSM, Diagnostic and Statistical Manual of Mental Disorders; EAS, Emotionality, Activity and Shyness Temperament Questionnaire; EEA, Environment of Evolutionary Adaptation; MoBa, Norwegian Mother and Child Cohort Study; SCARED, Screen for Child Anxiety Related Emotional Disorders; SMFQ, Short Mood and Feelings Questionnaire; SPSS, Statistical Package for the Social Sciences
